# Enface Thickness Mapping and Reflectance Imaging of Retinal Layers in Diabetic Retinopathy

**DOI:** 10.1371/journal.pone.0145628

**Published:** 2015-12-23

**Authors:** Andrew W. Francis, Justin Wanek, Jennifer I. Lim, Mahnaz Shahidi

**Affiliations:** Department of Ophthalmology and Visual Sciences, University of Illinois at Chicago, Chicago, Illinois, United States of America; National Eye Institute, UNITED STATES

## Abstract

**Purpose:**

To present a method for image segmentation and generation of enface thickness maps and reflectance images of retinal layers in healthy and diabetic retinopathy (DR) subjects.

**Methods:**

High density spectral domain optical coherence tomography (SDOCT) images were acquired in 10 healthy and 4 DR subjects. Customized image analysis software identified 5 retinal cell layer interfaces and generated thickness maps and reflectance images of the total retina (TR), inner retina (IR), outer retina (OR), and the inner segment ellipsoid (ISe) band. Thickness maps in DR subjects were compared to those of healthy subjects by generating deviation maps which displayed retinal locations with thickness below, within, and above the normal 95% confidence interval.

**Results:**

In healthy subjects, TR and IR thickness maps displayed the foveal depression and increased thickness in the parafoveal region. OR and ISe thickness maps showed increased thickness at the fovea, consistent with normal retinal anatomy. In DR subjects, thickening and thinning in localized regions were demonstrated on TR, IR, OR, and ISe thickness maps, corresponding to retinal edema and atrophy, respectively. TR and OR reflectance images showed reduced reflectivity in regions of increased thickness. Hard exudates appeared as hyper-reflective spots in IR reflectance images and casted shadows on the deeper OR and ISe reflectance images. The ISe reflectance image clearly showed the presence of focal laser scars.

**Conclusions:**

Enface thickness mapping and reflectance imaging of retinal layers is a potentially useful method for quantifying the spatial and axial extent of pathologies due to DR.

## Introduction

Spectral domain optical coherence tomography (SDOCT) has become an essential tool in the management of patients with diabetic retinopathy (DR) and has improved clinicians’ abilities to detect anatomical alterations in the retinal tissue as compared to conventional ophthalmoscopy or fundus photography.[1–10] Specifically, SDOCT B-scans allow depth-resolved visualization of pathologies resulting from non-proliferative diabetic retinopathy (NPDR), proliferative diabetic retinopathy (PDR), and diabetic macular edema (DME). These pathologies include diffuse retinal thickening and thinning, cystoid space formation, hard exudates, and tractional effects from abnormal neovascular tissue.[1–10]

SDOCT imaging visualizes the retinal tissue in axial cross-sections, permitting quantitative assessment of central submacular thickness, which is correlated with visual acuity in clinical treatment studies for DME.[11–19] The disadvantages of cross-sectional B-scans are limited visualization of the full spatial extent of irregular or large pathologies at different retinal layers and quantification of correlations between inner and outer retinal layer pathologies.

Recently developed image segmentation algorithms permit depth-resolved enface SDOCT imaging for viewing the retinal layers in the coronal plane. These enface SDOCT “C-scans” allow for quantifiable evaluation of individual retinal layers separated in depth. Several manual and semi-automated techniques have been developed for evaluation of the retinal layers in diabetics with the objective of improving the prognostic capability of SDOCT imaging.[20–26]

There is considerable evidence that the continuity of individual retinal layers has prognostic significance. Continuity of the inner segment ellipsoid (ISe) layer was found to correlate with visual acuity in several retinal conditions including DR.[27–40] Continuity of the ISe is determined by the relative brightness and thickness of this layer and may be affected by macular edema, ischemia or treatments including panretinal photocoagulation (PRP) or focal laser.[41, 42]

We have previously reported a method for generation of enface images of retinal layers from high-density SDOCT B-scans.[20, 43] Several groups have also published studies that apply enface SDOCT techniques for assessment of retinal diseases.[8, 38, 44–55] In the present study, we describe a method for semi-automated segmentation of retinal layers for generation of both enface thickness maps and reflectance images of the total retina, inner retina, outer retina, and the ISe layer in healthy control and DR subjects.

## Materials and Methods

### Subjects

Subjects were recruited from the ophthalmology clinics at the Illinois Eye and Ear Infirmary (University of Illinois at Chicago, Chicago, IL, USA). The research study was approved by an Institutional Review Board at the University of Illinois at Chicago. Prior to enrollment, the research study was explained to the subjects and written informed consents were obtained from each subject according to the tenets of the Declaration of Helsinki. SDOCT images were acquired in 10 healthy control subjects (age: 55 ± 8 years) and 4 DR subjects (age: 59 ± 12 years). The inclusion criterion for control subjects was no clinical history of diabetes or other retinal disease. SDOCT imaging was performed in one eye of each subject. Clinical data were obtained from the charts of DR subjects.

### Image Acquisition

A high density SDOCT raster volume scan of the macula was obtained using a commercially available instrument. (Spectralis; Heidelberg Engineering, Heidelberg, Germany). The volume scan consisted of 145 raster horizontal B-scans with a depth resolution of 3.9 μm and 768 A-scans per B-scan. The instrument’s eye tracking function allowed 9 B-scans to be averaged at each location. The SDOCT raster scan covered a retinal area of 15° x 15° centered on the fovea with approximately 31 micron spacing between SDOCT B-scans. Scanning laser ophthalmoscope (SLO) images were acquired with the same instrument.

### SDOCT Segmentation

Semi-automated image segmentation software was developed in MATLAB (Mathworks Inc., Natick, MA, USA) for identification of five interfaces between retinal cell layers in the SDOCT B-scans. Retinal cell layer interfaces were detected using graph theory and dynamic programming, based on a previous described method.[24] Briefly, a graph was created for each SDOCT B-scan with the edge weights of the graph assigned based on the vertical gradients in the image, such that large gradients resulted in small weights. A horizontal path through the graph that minimized the total sum of the weights was found using Dijkstra’s algorithm and defined a line separating two retinal cell layers.[24] By assigning weights of the graph according to the sign of the gradient (positive or negative), retinal cell layer interfaces that had bright to dark or dark to bright transitions were identified.

**Fig 1:**
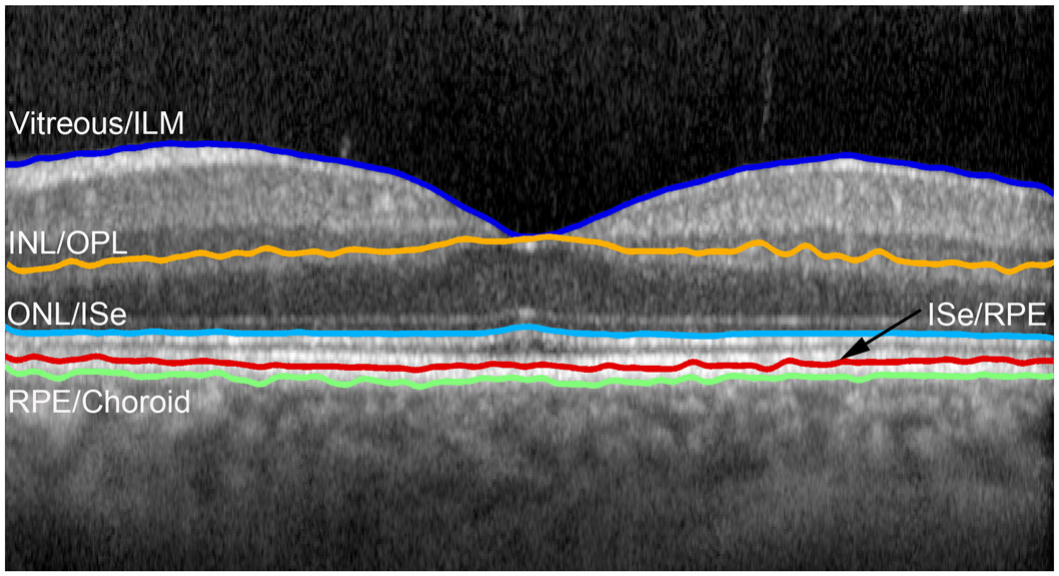
An example of a SDOCT B-scan image at the fovea in a healthy subject displaying segmentation of five retinal interfaces comprised of 1) vitreous and internal limiting membrane (ILM), 2) inner nuclear layer (INL) and outer plexiform layer (OPL), 3) outer nuclear layer (ONL) and inner segment ellipsoid (ISe), 4) ISe and retinal pigment epithelium (RPE), and 5) RPE and choroid.

As shown in Fig 1, the retinal interfaces detected were 1) vitreous and internal limiting membrane (ILM), 2) inner nuclear layer (INL) and outer plexiform layer (OPL), 3) outer nuclear layer (ONL) and inner segment ellipsoid (ISe), 4) ISe and retinal pigment epithelium (RPE), and 5) RPE and choroid. To find a unique path for these 5 retinal interfaces, image segmentation was performed in a set order. First, the interface between the vitreous and ILM was identified, since this interface was characterized by the largest dark to bright transition (largest positive vertical gradient) in the image and represented the lowest weighted path of the graph. Second, the interface between the ONL and ISe layers was found after restricting the graph search area to include only image regions below the vitreous/ILM path. Third, the path corresponding to the RPE/choroid interface was determined by restricting the graph search area to include only locations of the image below the ONL/ISe path and by assigning lower graph weights to larger negative gradients (bright to dark transition). Fourth, the INL/OPL cell interface was detected by limiting the graph to include only regions of the image between the vitreous/ILM and ONL/ISe paths. Finally, the ISe/RPE boundary was found by restricting the graph search area to include only image areas between the detected ONL/ISe and RPE/Choroid interface.

After automated segmentation of the retinal interfaces, the operator was able to scroll through all 145 SDOCT B-scans in the volume scan to review the segmentation results and, if necessary, manually correct errors in the detected interfaces. This process was necessary for images obtained in DR subjects due to the presence of irregular layer boundaries and intraretinal fluid. To correct segmentation errors, the operator would first select a segmentation path that required modification and then manually draw a revised line corresponding to the visualized cell layer interface. The search area of the graph was then restricted to include only a small vertical image region around the manually drawn line, and a revised path for the cell layer interface was obtained by determining a new graph cut solution.

### Enface Thickness Maps and Reflectance Images

Enface thickness maps and reflectance images were generated based on segmentation of the retinal interfaces in the SDOCT B-scans. Total retinal (TR) thickness was calculated as the depth separation between the vitreous/ILM and RPE/choroid interfaces. Inner retinal (IR) thickness was calculated as the depth separation between the vitreous/ILM and INL/OPL interfaces. Outer retina (OR) thickness was calculated as the depth separation between the INL/OPL and RPE/choroid interfaces. ISe thickness was calculated as the depth separation between the ONL/ISe and ISe/RPE interfaces. Enface reflectance images of TR, IR, OR, and ISe were generated based on pixel values averaged vertically within the segmented layers in each SDOCT B-scan to create rows of the corresponding enface images.

### Statistical Analysis

Thickness, standard deviation (SD), and 95% confidence interval (CI) maps for each segmented layer were generated from data in healthy subjects. Thickness maps obtained in DR subjects were compared to the normal CI maps using deviation maps. The deviation maps were generated by categorizing each pixel as below the lower limit, within the upper and lower limits, or above the upper limit of the normal CI and color coded as blue, green, and red, respectively.

## Results

### Control Subjects

**Fig 2:**
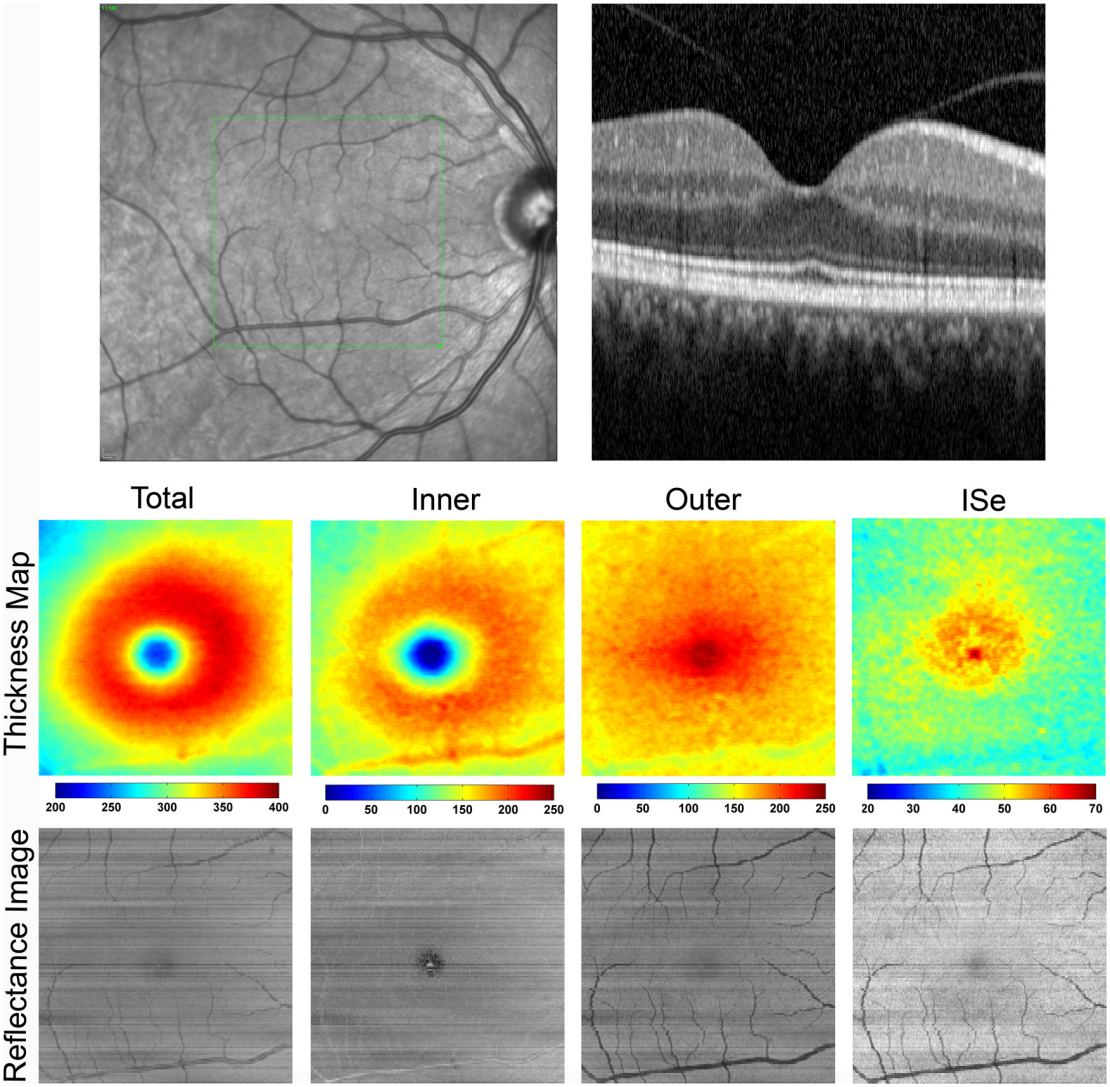
**Top**: SLO image with the corresponding SDOCT B-scan imaged through the fovea in the right eye of a control subject. **Bottom**: Thickness maps and reflectance images of the total retina, inner retina, outer retina, and the inner segment ellipsoid (ISe) layer are displayed. Color bars for thickness maps represent thickness in microns. Thickness maps were consistent with normal retinal anatomy. Outer retina and ISe reflectance images display shadowing of the retinal vasculature.

Fig 2 displays a SLO image showing the scanned retinal area, a SDOCT B-scan, enface thickness maps, and reflectance images for the right eye of a control subject. The TR thickness map displayed a central region of decreased thickness corresponding to the foveal depression, surrounded by a ring of increased thickness in the parafoveal region. Similarly, the IR thickness map showed a smooth, gradual decrease in thickness from the parafovea to the fovea as the inner retinal layers disappear. The OR thickness map demonstrated a gradual increase in thickness towards the foveal center due to the longer, more densely packed cones. Similarly, the ISe thickness map showed increased thickness centrally, because of a greater separation between the ellipsoid of the inner segments and the apical RPE at the fovea.[27]

Reflectance images displayed homogenous patterns without significant variations. The normal retinal vasculature including the major retinal arcades are marginally visible within the IR reflectance image, but appeared more prominently in the OR and ISe reflectance images due to shadowing effects. The foveal center appeared darker, relative to the surrounding parafoveal retina in reflectance images, but more prominently in the IR reflectance image due to the absence of inner retinal cell layers.

### Diabetic Subjects

#### Case 1: PDR and DME

A 42 year old female with a history of PDR and DME presented for follow up evaluation. She had previously received PRP for PDR, focal laser for DME, and injections of anti-vascular endothelial growth factor (anti-VEGF) agents in her left eye. Her vision was 20/25 and stable from previous visits. She had no new visual complaints. Fundus examination was notable for a blunted fovea, focal laser scars, and prominent macular edema.

**Fig 3:**
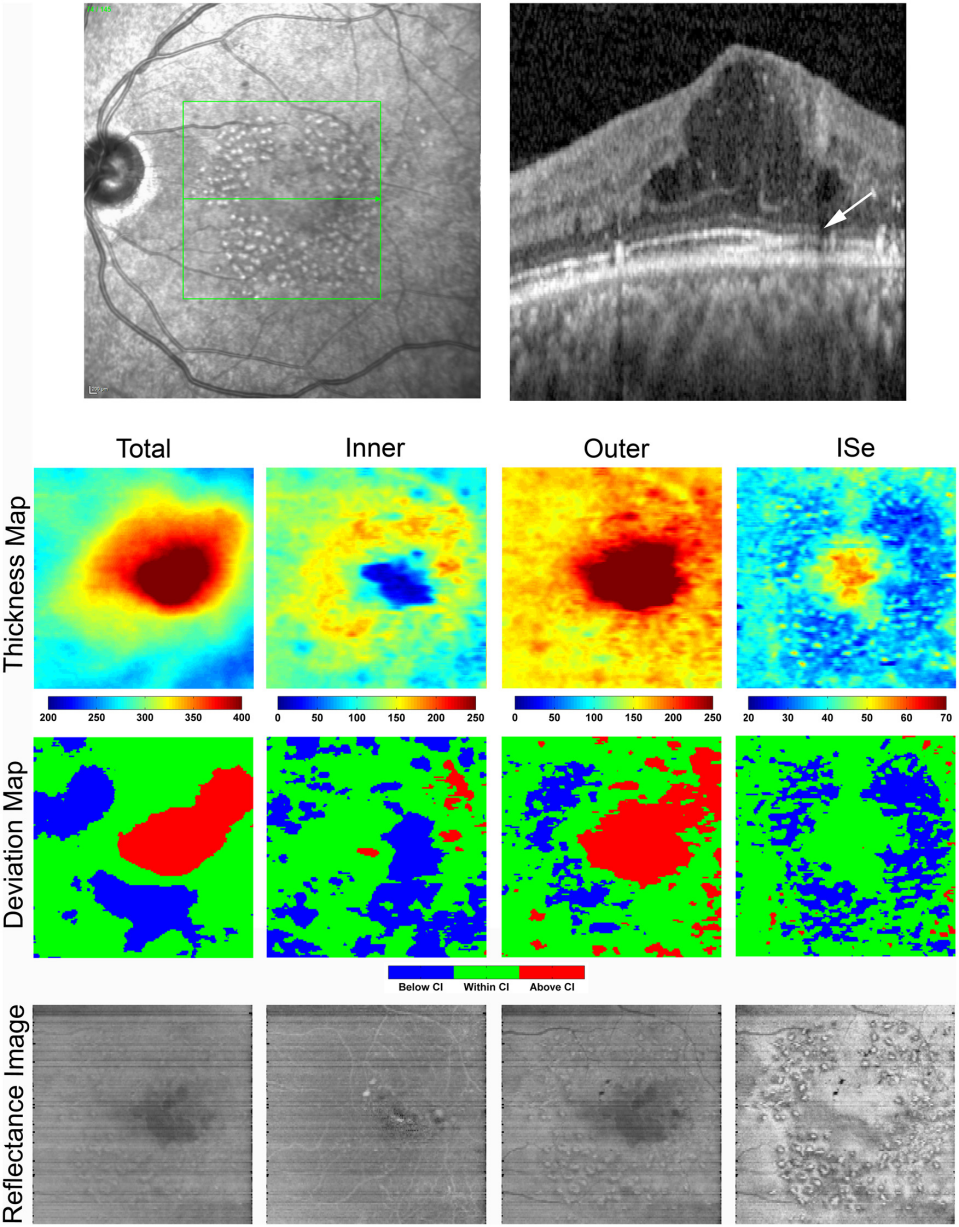
**Top**: SLO image with the corresponding SDOCT B-scan imaged through the fovea in the left eye of a DR subject with a history of PDR and DME (Case 1). The SLO image shows focal laser scars and the SDOCT B-scan displays cystoid DME, hard exudates, and inner segment ellipsoid (ISe) discontinuity (arrow). **Bottom**: Total retina (TR) and outer retinal (OR) thickness and deviation maps display central retinal thickening. The inner retina (IR) thickness map shows a distorted foveal depression and the inner segment ellipsoid (ISe) thickness map shows a ring-shaped area of thinning which is consistent with the pattern of focal laser scars seen in the SLO image. Color bars for thickness maps represent thickness in microns. TR and OR reflectance images show reduced reflectivity in regions of increased thickness. Hard exudates appear as hyper-reflective spots in the IR reflectance image and cast shadows on the deeper OR and ISe reflectance images. The ISe reflectance image clearly shows the presence of focal laser scars.

Fig 3 displays a SLO image showing the scanned retinal area, a SDOCT B-scan, enface thickness and deviation maps, and reflectance images obtained in the left eye. TR thickness and deviation maps showed foveal and parafoveal regions of retinal thickening. Thickening due to cystoid DME was predominantly located in the OR and to a lesser degree in the IR, as shown in OR thickness and deviation maps as compared to the IR maps. The IR thickness map was notable for a distorted foveal depression that was also depicted in the IR deviation map. The ISe thickness map showed increased thickness centrally, similar to control subjects, accounting for the unaffected visual acuity. The ISe thickness and deviation maps displayed a ring-shaped area of thinning which was consistent with the pattern of focal laser scars seen in the SLO image.

Both the TR and OR reflectance images displayed regions of reduced reflectivity that corresponded to regions of increased retinal thickness. The IR reflectance image showed a region of reduced reflectivity near the distorted foveal depression. Hard exudates were visible in the IR reflectance image as hyper-reflective spots that cast shadows on the deeper OR and ISe reflectance images. Multiple focal laser scars were visible in the ISe reflectance image.

#### Case 2: PDR and DME

A 62 year old female with a history of PDR with DME presented for evaluation. She had previously received focal laser for DME in addition to several anti-VEGF injections in her left eye. Visual acuity was 20/100. Fundus examination was notable for the presence of significant retinal thickening supero-nasal to the fovea, consistent with clinically significant DME.

**Fig 4:**
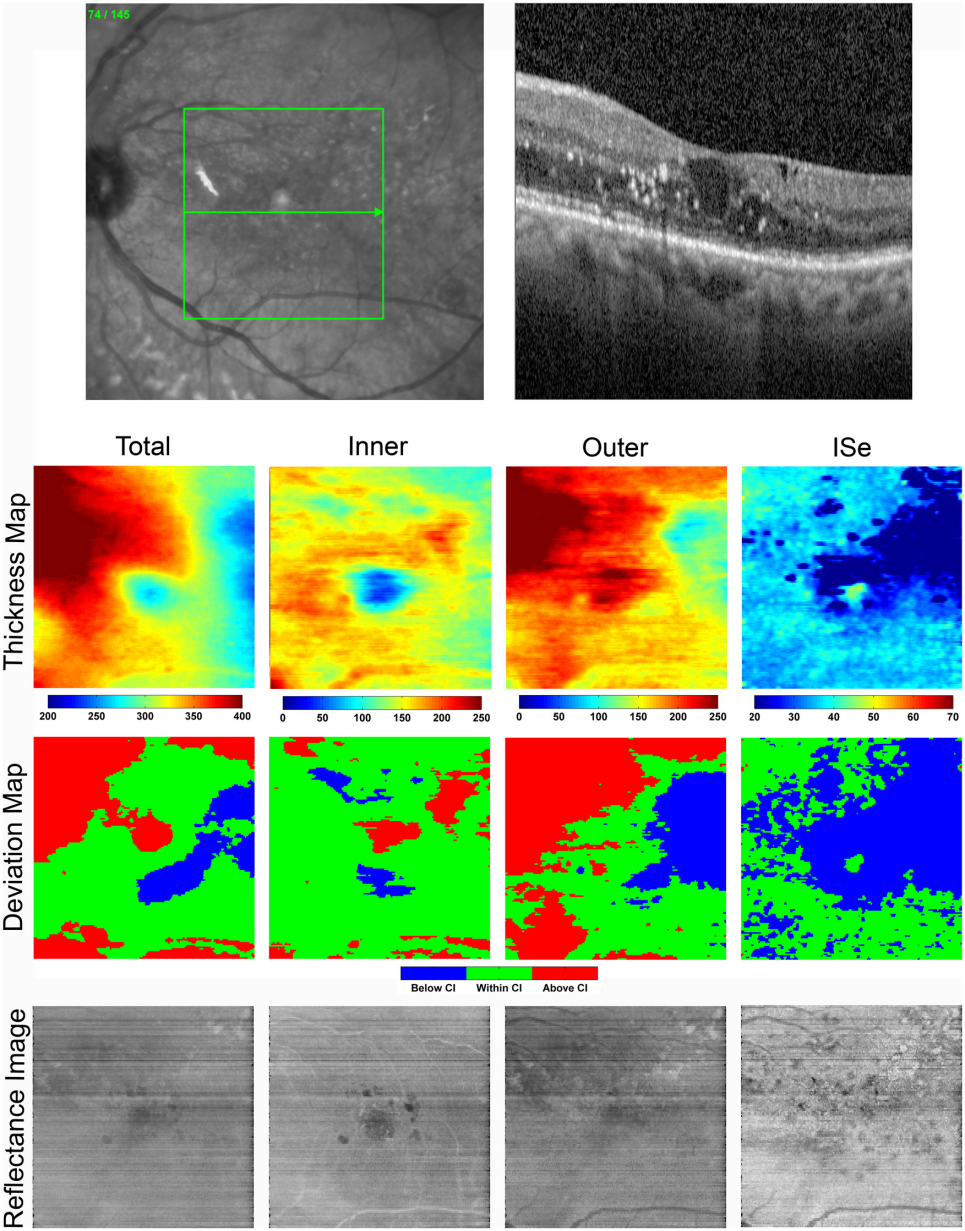
**Top**: SLO and SDOCT B-scan in a PDR subject with a history of DME in the left eye (Case 2). SDOCT B-scan shows irregular retinal architecture, hard exudates, and cystoid DME at the fovea. **Bottom**: Total retina (TR) and outer retina (OR) thickness and deviation maps reveal substantial regions of retinal thickening. The inner segment ellipsoid (ISe) thickness map shows thinning supero-temporal to the fovea. Color bars for thickness maps represent thickness in microns. The ISe reflectance image displays spots of reflectance inhomogeneity due to laser scars in the same region.

Fig 4 displays a SLO image showing the scanned retinal area, a SDOCT B-scan, enface thickness and deviation maps, and reflectance images obtained in the left eye. The SDOCT B-scan demonstrated irregular retinal architecture with a large cystoid macular edema at the fovea and hard exudate formation in parafoveal areas. TR and OR thickness and deviation maps showed substantial thickening in supero-nasal areas. Foveal thickening was present in the TR and IR thickness maps corresponding to cystoid macular edema observed on the SDOCT B-scan through the fovea. The ISe thickness map demonstrated thinning supero-temporal to the fovea in a region heavily affected by focal laser scars. On the OR reflectance image, dark areas located supero-nasal to the fovea corresponded with regions of increased TR and OR thickness. Spots of reflectance inhomogeneity supero-temporal to the fovea on the ISe reflectance image were consistent with focal laser scars and corresponded to a large region of ISe thinning, as visualized on the ISe thickness map.

#### Case 3: PDR, DME and atrophy

A 66 year old male with a history of PDR and DME in his left eye presented for follow up evaluation. He had a history of PRP treatment and had undergone focal laser therapy in the left eye for DME 3 months prior to this visit. Visual acuity was 20/40 and he had no new visual complaints.

**Fig 5:**
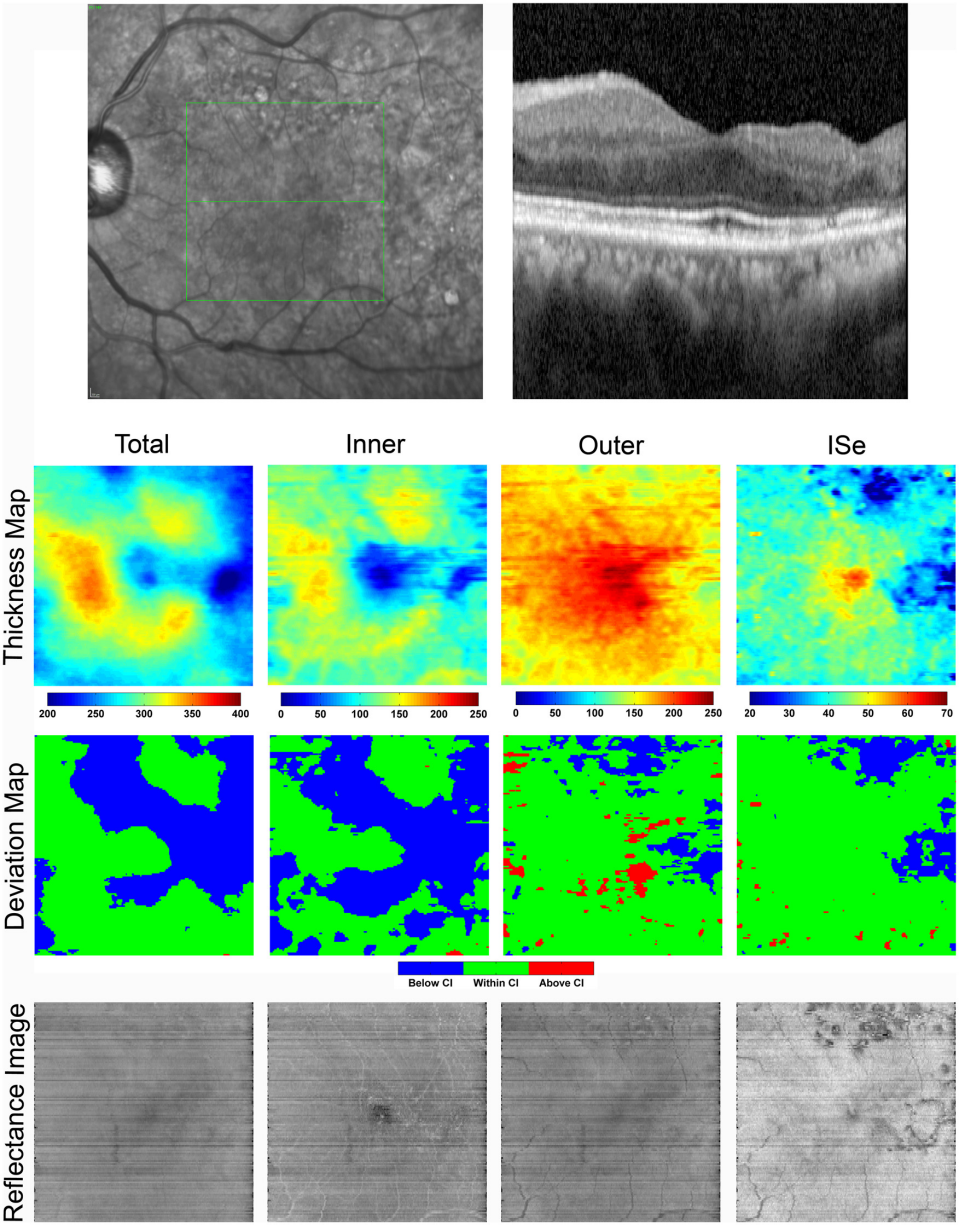
**Top**: SLO and SDOCT B-scan images in a PDR subject with a history of DME and retinal atrophy (Case 3). The SDOCT B-scan shows areas of inner retinal (IR) thinning with preservation of the outer retina (OR) and inner segment ellipsoid (ISe) centrally. **Bottom:** Total retina (TR) and IR thickness and deviation maps showed large regions of retinal thinning. The OR and ISe thickness maps demonstrate preservation of the normal retinal anatomy near the fovea with scattered areas of thinning in the periphery, due to focal laser scars. Color bars for thickness maps represent thickness in microns. OR and ISe reflectance images display scattered focal hypo-reflective spots due to laser scars superiorly and temporally.

Fig 5 displays a SLO image showing the scanned retinal area, a SDOCT B-scan, enface thickness and deviation maps, and reflectance images obtained in the left eye. The SDOCT B-scan showed areas of inner retinal thinning with preservation of the OR and ISe centrally. TR and IR thickness maps demonstrate large regions of retinal thinning indicated by the deviation maps. The OR thickness map demonstrated the overall preservation of normal retinal anatomy, except for local regions of abnormal thinning and thickening, as shown in the OR deviation map. The ISe thickness map shows reduced thickness superiorly and temporally, but normal thickness near the fovea. IR and OR reflectance images were relatively homogenous. Scattered focal hypo-reflective spots due to laser scars were observed superiorly and temporally on the ISe reflectance image, corresponding to localized thinning.

#### Case 4: NPDR and DME

A 61 year old male with a history of NPDR and DME in his left eye presented for follow up examination. His visual acuity was 20/20 and he had no new visual complaints. Fundus ophthalmoscopy initially identified an area of increased retinal thickening nasal to the fovea.

**Fig 6:**
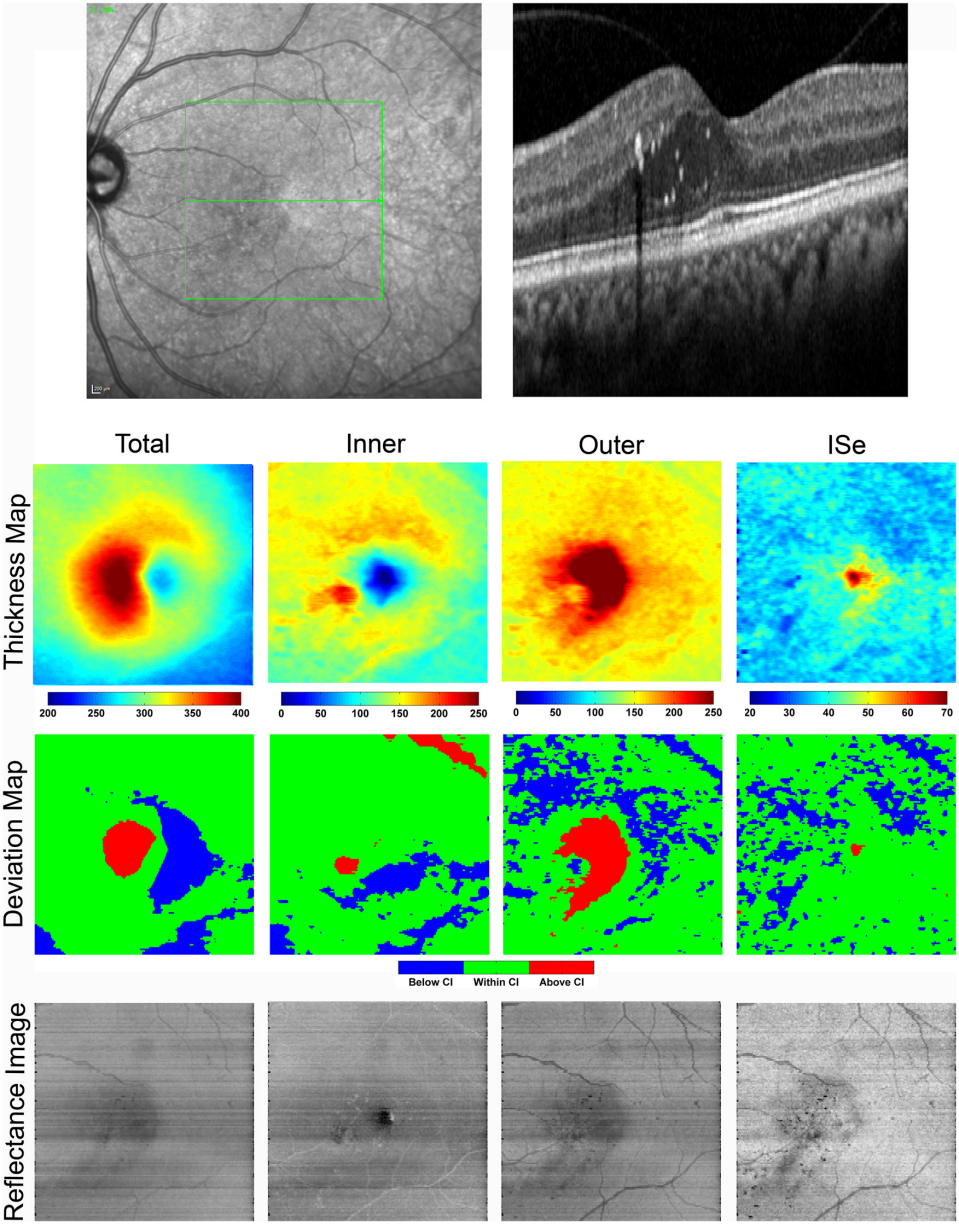
**Top**: SLO and SDOCT B-scans of the left eye in a NPDR subject with a history of DME (Case 4). On the SDOCT B-scan, there is a temporal region of cystoid DME and hard exudates in the outer retina (OR) resulting in distortion of the foveal depression. The inner segment ellipsoid (ISe) layer appears intact. **Bottom**: Total retina (TR) and OR thickness and deviation maps show thickening at the fovea. The inner retina (IR) thickness and deviation maps show preservation of the normal foveal depression with nonspecific scattered areas of thinning inferiorly. ISe thickness and deviation maps show a central region of increased thickness that corresponds to the normal anatomy. Color bars for thickness maps represent thickness in microns. TR and OR reflectance images show reduced reflectivity in focal regions of increased thickness. The IR reflectance image demonstrates scattered hyper-reflectance spots from hard exudates. The ISe reflectance image shows hypo-reflectance spots that correspond to shadows from hard exudates in the inner retina.

Fig 6 displays a SLO image showing the scanned retinal area, a SDOCT B-scan, enface thickness and deviation maps, and reflectance images obtained in the left eye. On the SDOCT B-scan, cystoid DME and retinal thickening near the fovea was present. TR and OR thickness and deviation maps showed a region of increased retinal thickness. The IR thickness map showed preservation of the foveal depression with nonspecific scattered areas of thinning. The ISe thickness map displayed a central foveal region of increased thickness that corresponded to the normal anatomy.

TR and OR reflectance images showed a diffuse area of reduced reflectivity in the region of increased thickness. The IR reflectance image demonstrated scattered areas of hyper-reflectance from hard exudates. The ISe reflectance image showed hypo-reflective spots that correspond to shadows from hard exudates in the inner retina.

## Discussion

The current study reports a method for generating enface thickness maps and reflectance images of retinal layers using a commercially available SDOCT instrument. The method established normal retinal anatomical features in TR, OR, IR, and ISe thickness maps and reflectance images, and detected pathologic alterations in selected cases of DR. In subjects with DR, pathologic alterations, including thickening and thinning of TR, IR, OR, and ISe across the macular area, were quantitatively displayed on the enface thickness and deviation maps. Furthermore, reflectance images of TR, IR, OR, and ISe displayed regions of reduced reflectivity that corresponded with areas of increased thickness and disruption of ISe integrity. Hard exudates were consistently observed as hyper-reflective regions on IR reflectance images and hypo-reflective regions on OR and ISe reflectance images.

Several previous studies have reported methods for segmentation of different retinal layers in DR.[22–26, 43, 56] Mohammad et al[43] proposed an automated level-set method for co-localization of pathologies in enface images of retinal layers. Huang et al[22] utilized an EdgeSelect method to perform semi-automated segmentation of retinal layers in DR subjects. Chiu et al[23, 24, 56] developed an automated algorithm to identify eight retinal layer boundaries on SDOCT images in DME subjects. In addition, the commercial Heidelberg Eye Explorer software has recently provided automated layer segmentation for generation of enface thickness maps and reflectance images of retinal layers. However, this software has built-in retinal layer boundaries for thickness mapping that cannot be modified by the user, such as specifying the external limiting membrane as the interface between inner and outer retina. Furthermore, accurate automated segmentation of retinal layers is complicated by the heterogeneous presentation of DR. Boundaries between retinal layers in subjects with distorted retinal architecture from cystoid DME are often poorly detected by computer algorithms, necessitating user interaction. The semi-automated segmentation method presented in the current study was capable of effectively identifying retinal layer boundaries and generating both enface thickness maps and reflectance images. Future studies comparing segmentation of individual retinal layers by different image analysis techniques are warranted.

Studies employing segmentation techniques to investigate reflectance changes of individual, depth resolved retinal layers in an enface manner are lacking. Murakami et al[37] investigated the relationship between diabetic cystoid spaces and the characteristics of the photoreceptors beneath the cystoid spaces in DME subjects. They reported that areas beneath the cystoid spaces had greater disruption of the ISe than areas without cystoid spaces. In the current study, enface reflectance images of the ISe layer were generated, providing a more accurate and localized assessment of the ISe integrity. Further studies investigating reflectance variations in different retinal layers in DR subjects are needed.

In the current study, DR subjects who maintained good visual acuity had ISe reflectance at the fovea similar to that of control subjects. Although additional subjects are needed to establish a correspondence between enface ISe reflectance images and visual acuity, this finding is in agreement with previous studies that showed ISe layer continuity is correlated with visual acuity. [28, 30–33, 57] Because thickness maps and reflectance images are capable of accurately displaying foveal pathology, any alteration in this region may be clinically relevant and may precede detectable findings on clinical examination. Additionally, restoration of the ISe layer may indicate resolving pathology. Thickness maps and reflectance images can also be overlaid and combined for accurate comparison between clinical visits, allowing for detection of subtle alterations in retinal anatomy.

Enface OCT imaging has several practical advantages compared to standard cross-sectional OCT imaging. Enface OCT images generated from a high density raster of OCT B-scans enable detection of subtle retinal pathology that may fall between standard OCT B-scans. Additionally, enface OCT imaging allows succinct visualization of the spatial extent of pathologies in different retinal layers compared to scrolling through individual OCT B-scans. This feature is particularly useful for accurate monitoring of pathological changes over time. Enface OCT imaging may also be an effective method to study changes in choroidal thickness, which have been associated with disease severity in DR, although this was not assessed in the current study. Based on individual OCT B-scans, choroidal thickness was reported to be reduced in eyes with moderate-to-severe DR and DME compared to age-matched controls.[58, 59] Additionally, choroidal thickness mapping showed eyes with microaneurysms, hard exudates and DME had a significantly thinner choroid compared to age-matched controls.[60, 61] This suggests the pathogenesis of DR may be associated with a choroidal angiopathy, in addition to microvascular changes in the inner retina. Furthermore, OCT angiography is now available for generating enface images of the retinal vasculature. Combined enface assessment of the retinal anatomy and vasculature would provide a comprehensive means for clinical assessment of DR.

In summary, the reported method for segmentation of SDOCT B-scan images and generation of enface thickness maps and reflectance images of retinal layers has the advantage of quantifying the spatial and axial extent of DR pathologies. Future studies are necessary to investigate the clinical utility of enface thickness mapping and reflectance imaging in DR and other retinal diseases.
